# Supplementary material for: Neutralization against Omicron SARS-CoV-2 from previous non-Omicron infection
Source: Nat Commun. 2022 Feb 9;13:852. doi: 10.1038/s41467-022-28544-w (PMC8828871; doi:10.1038/s41467-022-28544-w)
Supplement: Supplementary file 1 — Supplementary information [file 41467_2022_28544_MOESM1_ESM.pdf]

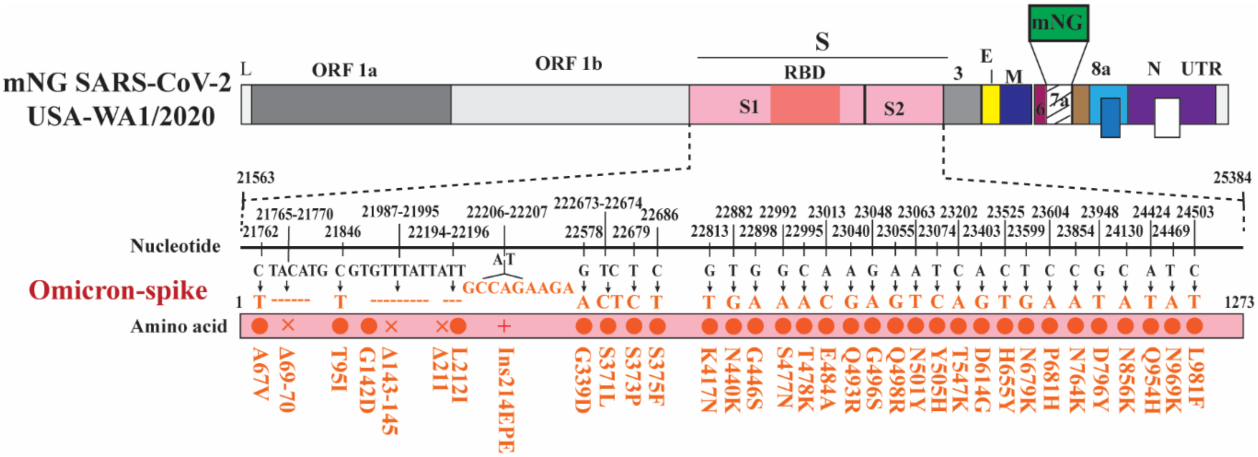


**Supplementary Figure 1**. Construction of mNeonGreen (mNG) Omicron-spike SARS-CoV-2. mNG USA-WA1/2020 was used to engineer the complete *spike* gene from the Omicron variant, resulting in mNG Omicron-spike SARS-CoV-2. Mutations (red circle), deletions (x), and insertions (+) are indicated. Nucleotide and amino acid positions are depicted. L: leader sequence; ORF: open reading frame; RBD: receptor binding domain; S: spike glycoprotein; S1: N-terminal furin cleavage fragment of S; S2: C-terminal furin cleavage fragment of S; E: envelope protein; M: membrane protein; N: nucleoprotein; UTR: untranslated region.

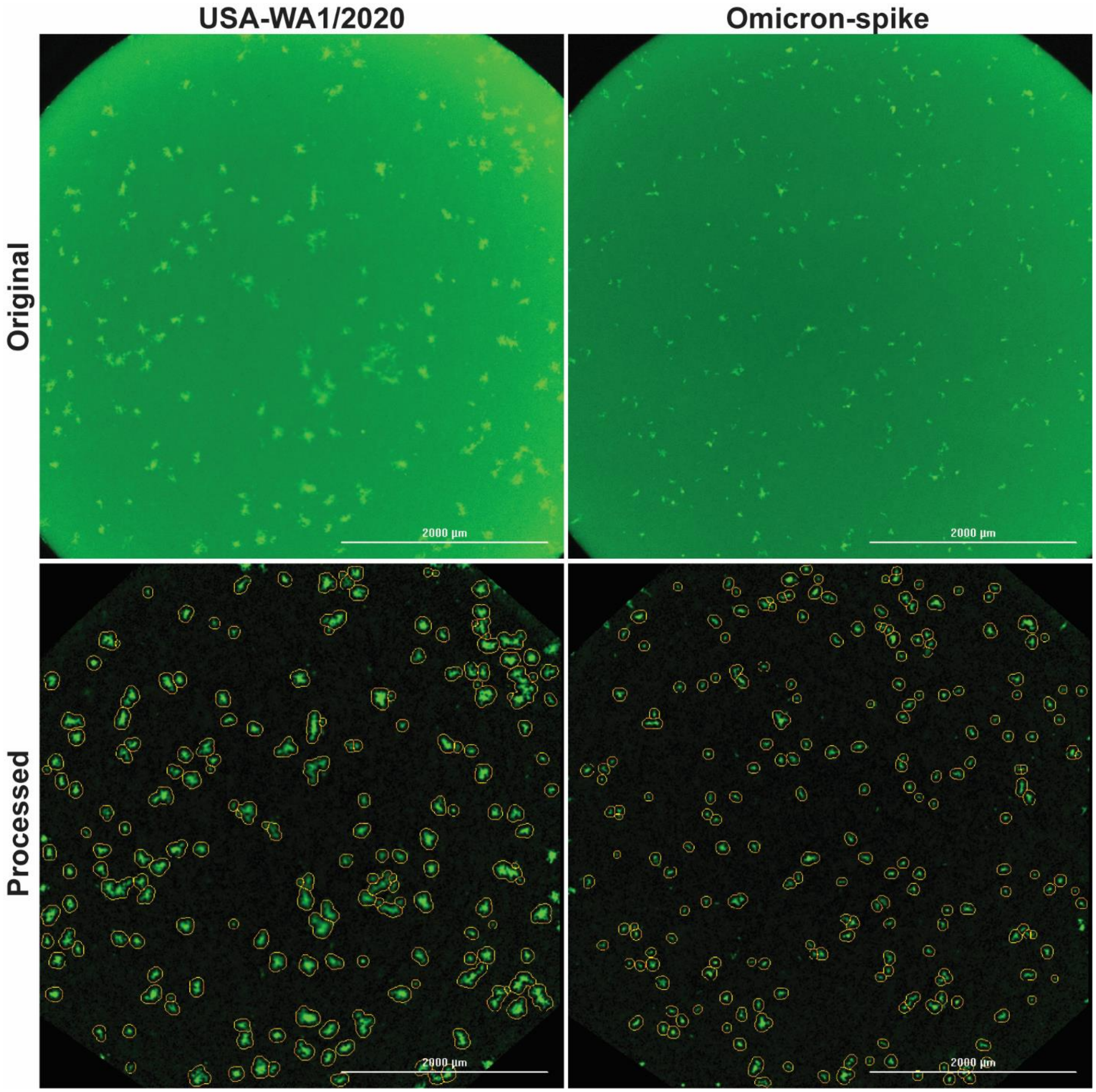


**Supplementary Figure 2**. Fluorescent foci of mNG USA-WA1/2020 and mNG Omicron-spike SARS-CoV-2 on Vero E6 cells. Original and processed images were collected by high-content imaging. The protocol of the fluorescent focus reduction neutralization test (FFRNT) is described in Methods. See **Supplementary Figure 3** for the experimental scheme of FFRNT.

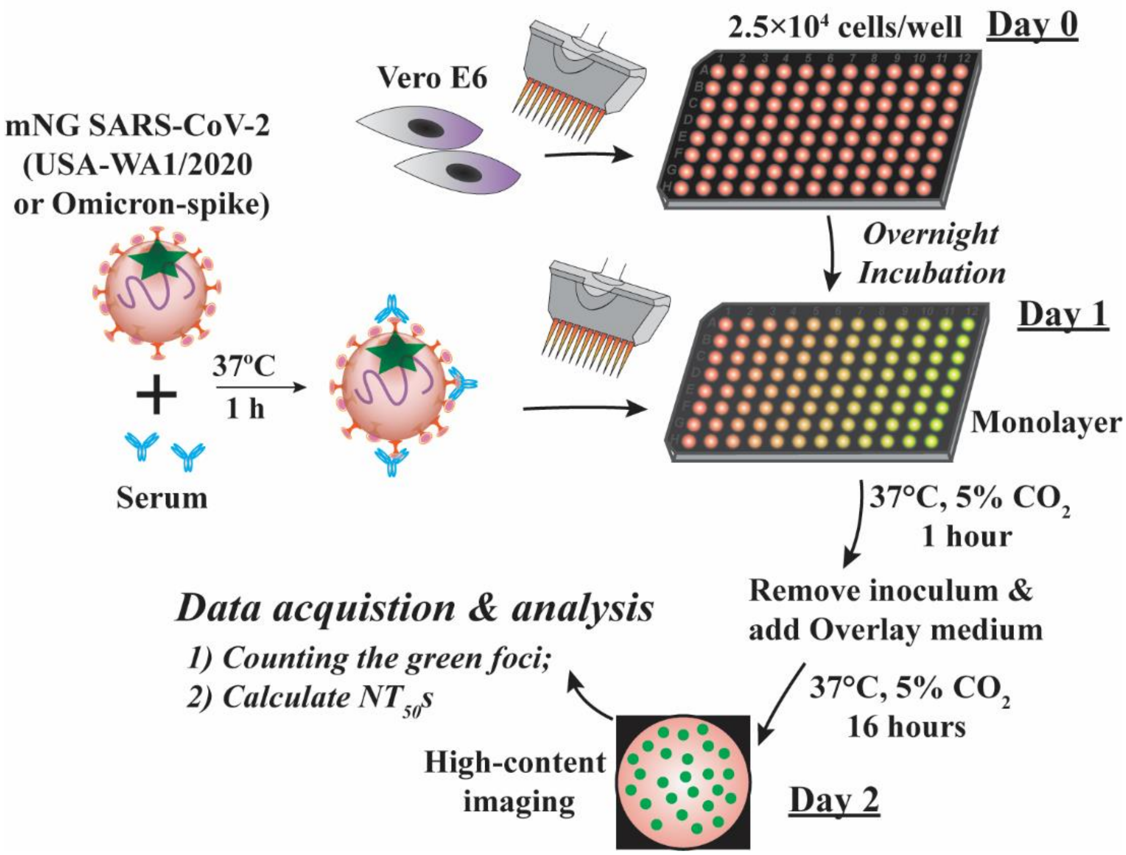


**Supplementary Figure 3**. Experimental scheme of fluorescent focus reduction neutralization test (FFRNT). The FFRNT protocol is described in Methods.

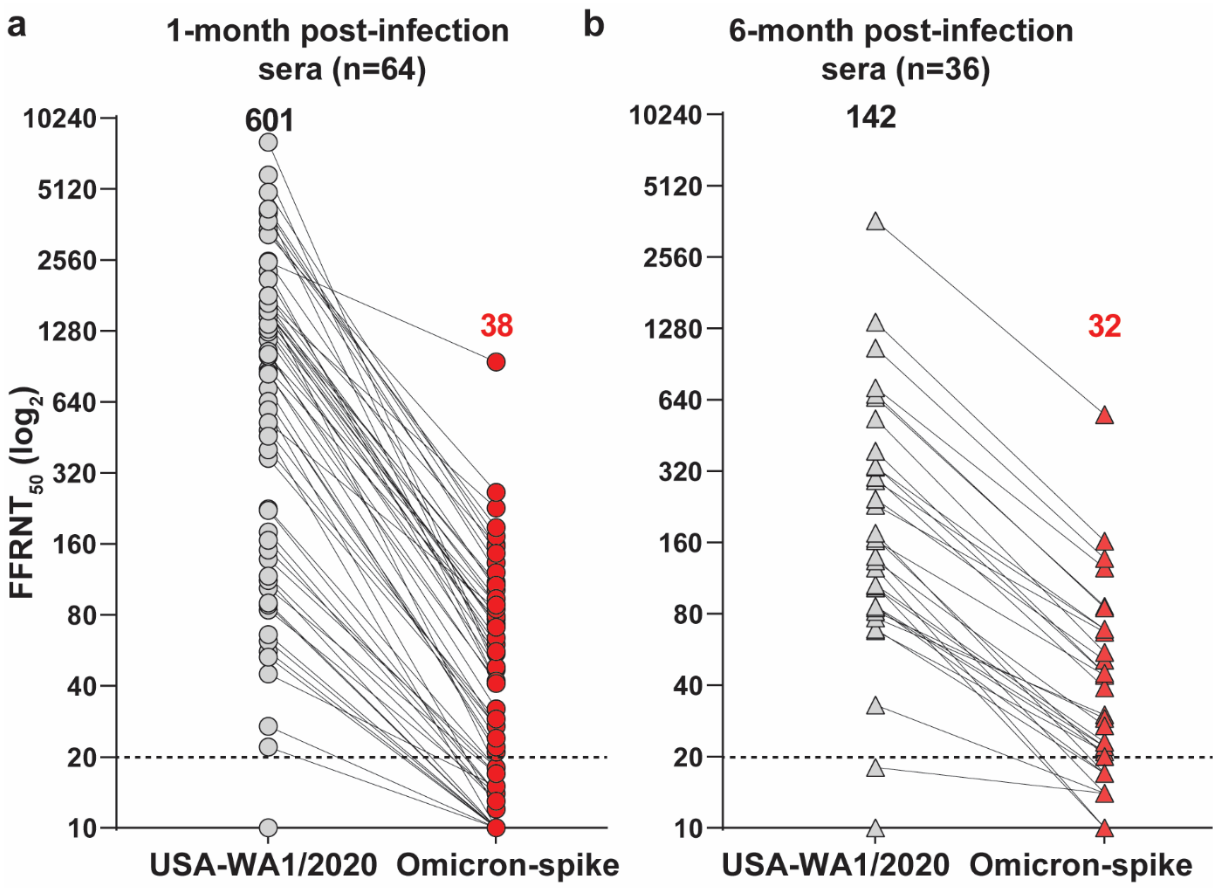


**Supplementary Figure 4**. Reduced neutralization against Omicron SARS-CoV-2 by previous non-Omicron viral infection. 50% fluorescent focus reduction neutralization titers ($FFRNT_{50}$) were measured for two serum panels from COVID-19 patients previously infected with non-Omicron SARS-CoV-2. The first serum panel was collected at 1-month post-infection (n=64) and the second panel collected at 6-month post-infection (n=36). For each serum, two $FFRNT_{50}$ values against mNG USA-WA1/2020 and Omicron-spike SARS-CoV-2 are connected by a line. **a,** $FFRNT_{50}$s of 1-month post-infection sera. **b,** $FFRNT_{50}$s of 6-month post-infection sera. **Extended Data Tables 1** and **2** summarize the $FFRNT_{50}$ values and serum information for (**a**) and (**b**), respectively. This figure is a reformat of **Figure 1**.

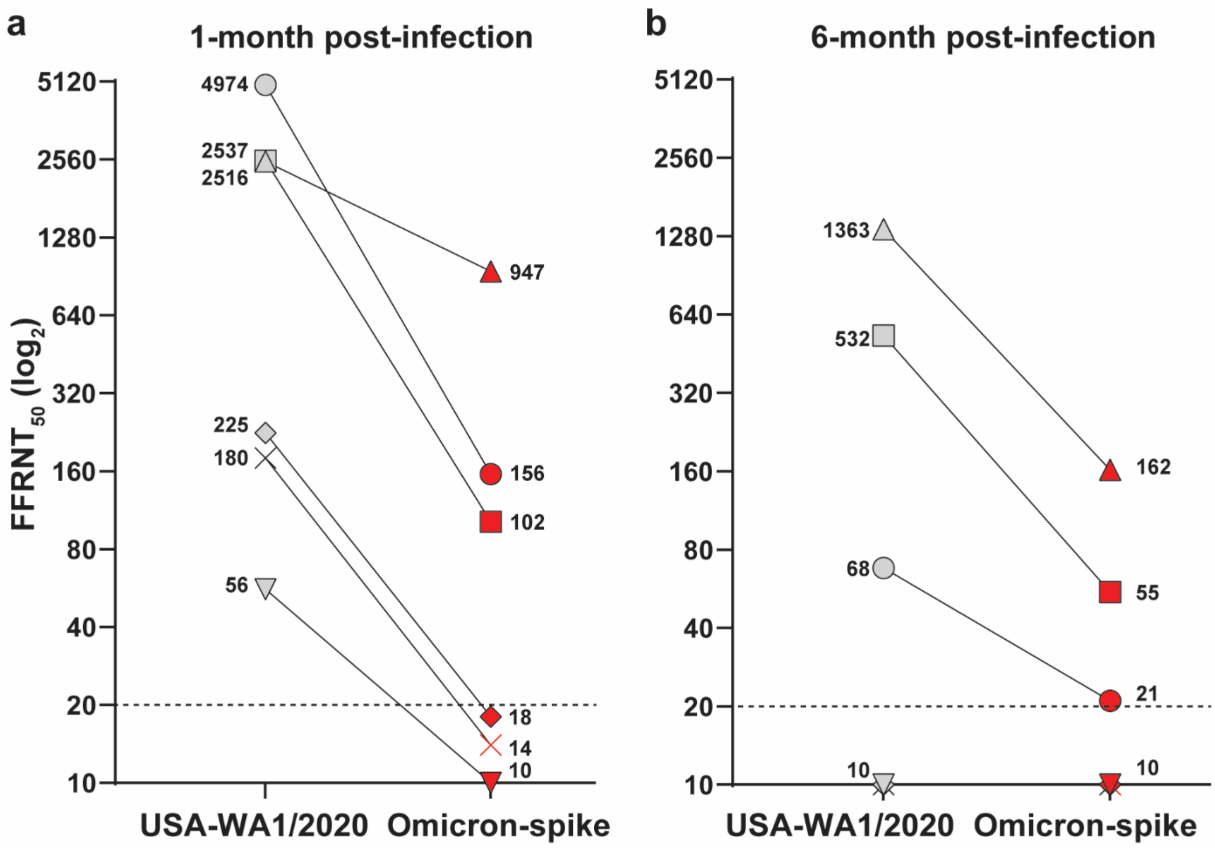


**Supplementary Figure 5**. FFRNT$_{50}$ of 6 pairs of 1- and 6-month post-infection sera from same patients. **a,** FFRNT$_{50}$s of 1-month post-infection sera against mNG USA-WA1/2020 and Omicron-spike SARS-CoV-2. **b,** FFRNT$_{50}$s of 6-month post-infection sera against mNG USA-WA1/2020 and Omicron-spike SARS-CoV-2.

**Supplementary Table 1.** $FFRNT_{50}$ values of 1-month post-infection sera against mNG USA-WA1/2020 and Omicron-spike SARS-CoV-2

| Serum ID | Age | Gender | Race and Ethnicity | Sample collection date yielding positive viral test | Symptomatic | Hospitalized | $FFRNT_{50}$ | |
|---|---|---|---|---|---|---|---|---|
| | | | | | | | USA-WA1/2020 | Omicron-spike |
| 1 | 21 | F | Hispanic or Latino | 7/22/2020 | No | No | 10 | 10 |
| 2 | 38 | F | White | 11/27/2020 | No | No | 22 | 10 |
| 3 | 17 | M | Hispanic or Latino | 6/1/2020 | Yes | No | 27 | 10 |
| 4 | 18 | F | Hispanic or Latino | 7/11/2020 | Yes | No | 45 | 15 |
| 5 | 26 | F | Hispanic or Latino | 11/11/2020 | Yes | No | 53 | 10 |
| ▽ 6 | 24 | F | Hispanic or Latino | 6/24/2020 | No | No | 56 | 10 |
| 7 | 24 | F | Hispanic or Latino | 7/27/2020 | Yes | No | 62 | 10 |
| 8 | 35 | F | Hispanic or Latino | 1/5/2021 | No | No | 66 | 10 |
| 9 | 23 | F | Hispanic or Latino | 6/25/2020 | No | No | 84 | 14 |
| 10 | 24 | F | Hispanic or Latino | 7/2/2020 | Yes | No | 88 | 10 |
| 11 | 33 | F | Black or African American | 11/21/2020 | Yes | No | 90 | 10 |
| 12 | 26 | F | Hispanic or Latino | 7/2/2020 | Yes | No | 104 | 17 |
| 13 | 60 | M | White | 9/26/2020 | Yes | Yes | 112 | 10 |
| 14 | 67 | M | Caucasian/White | 11/16/2020 | Yes | No | 117 | 13 |
| 15 | 22 | M | Hispanic or Latino | 9/24/2020 | No | No | 138 | 18 |
| 16 | 69 | M | White | 12/28/2020 | Yes | No | 151 | 17 |
| 17 | 73 | M | White | 5/27/2020 | Yes | No | 166 | 13 |
| × 18 | 17 | F | Hispanic or Latino | 6/22/2020 | Yes | No | 180 | 14 |
| 19 | 45 | M | Hispanic or Latino | 5/2/2020 | Yes | Yes | 222 | 14 |
| ◇ 20 | 24 | F | Hispanic or Latino | 6/24/2020 | Yes | No | 225 | 18 |
| 21 | 25 | F | Black or African American | 7/16/2020 | No | No | 369 | 27 |
| 22 | 75 | F | Hispanic or Latino | 9/14/2020 | Yes | No | 402 | 29 |
| *23 | 80 | F | White | 11/9/2020 | Yes | Yes | 459 | 10 |
| 24 | 61 | F | White | 11/3/2020 | Yes | No | 486 | 27 |
| 25 | 78 | M | White | 12/15/2020 | Yes | No | 524 | 85 |
| 26 | 66 | M | White | 5/30/2020 | Yes | Yes | 592 | 12 |
| 27 | 38 | M | Hispanic or Latino | 6/27/2020 | No | No | 640 | 24 |
| 28 | 34 | M | Hispanic or Latino | 11/11/2020 | Yes | No | 645 | 22 |
| 29 | 25 | F | Hispanic or Latino | 7/17/2020 | No | No | 730 | 21 |
| 30 | 66 | M | Black or African American | 4/27/2020 | Yes | Yes | 840 | 42 |
| 31 | 39 | M | Black or African American | 4/9/2020 | Yes | Yes | 856 | 27 |
| 32 | 35 | F | Hispanic or Latino | 10/15/2020 | Yes | Yes | 882 | 77 |

| | | | | | | | | |
|---|---|---|---|---|---|---|---|---|
| 33 | 55 | M | Hispanic or Latino | 5/6/2020 | Yes | Yes | 1003 | 89 |
| *34 | 67 | F | Hispanic or Latino | 1/4/2021 | Yes | Yes | 1020 | 41 |
| 35 | 55 | F | White | 9/28/2020 | Yes | No | 1050 | 32 |
| 36 | 40 | F | Hispanic or Latino | 12/20/2020 | Yes | No | 1174 | 55 |
| 37 | 60 | F | Hispanic or Latino | 11/27/2020 | Yes | Yes | 1268 | 48 |
| 38 | 65 | M | Hispanic or Latino | 5/7/2020 | Yes | Yes | 1306 | 64 |
| *39 | 69 | M | White | 11/22/2020 | Yes | Yes | 1365 | 79 |
| 40 | 68 | M | Caucasian/White | 5/10/2020 | Yes | Yes | 1454 | 73 |
| 41 | 50 | M | Black or African American | 4/8/2020 | Yes | Yes | 1465 | 60 |
| 42 | 63 | M | Hispanic or Latino | 1/23/2021 | Yes | Yes | 1517 | 94 |
| 43 | 39 | M | Black or African American | 3/31/2020 | Yes | Yes | 1519 | 110 |
| 44 | 72 | M | White | 12/23/2020 | Yes | Yes | 1555 | 73 |
| 45 | 55 | F | White | 10/6/2020 | Yes | Yes | 1584 | 107 |
| 46 | 57 | M | White | 7/5/2020 | Yes | No | 1618 | 18 |
| 47 | 1 | F | Hispanic or Latino | 1/18/2021 | Yes | Yes | 1638 | 227 |
| 48 | 87 | M | White | 1/5/2021 | Yes | Yes | 1679 | 71 |
| 49 | 96 | F | White | 12/30/2020 | Yes | Yes | 1807 | 88 |
| 50 | 66 | M | Hispanic or Latino | 12/19/2020 | Yes | No | 1814 | 159 |
| 51 | 75 | M | Hispanic or Latino | 10/27/2020 | Yes | No | 2119 | 56 |
| 52 | 63 | F | Hispanic or Latino | 12/12/2020 | Yes | Yes | 2289 | 42 |
| △53 | 66 | M | White | 12/27/2020 | Yes | Yes | 2516 | 947 |
| □54 | 49 | M | Black or African American | 1/3/2021 | No | Yes | 2537 | 102 |
| 55 | 56 | M | Hispanic or Latino | 7/13/2020 | Yes | Yes | 3277 | 265 |
| 56 | 44 | F | Black or African American | 8/20/20/ | Yes | Yes | 3443 | 133 |
| *57 | 83 | M | Hispanic or Latino | 11/22/2020 | Yes | Yes | 3464 | 188 |
| *58 | 75 | M | White | 12/27/2020 | Yes | Yes | 3741 | 172 |
| *59 | 74 | M | White | 12/21/2020 | Yes | Yes | 4055 | 42 |
| 60 | 48 | F | Hispanic or Latino | 6/21/2020 | Yes | Yes | 4116 | 121 |
| 61 | 78 | F | Hispanic or Latino | 12/16/2020 | Yes | Yes | 4216 | 146 |
| ○*62 | 70 | M | Hispanic or Latino | 12/12/2020 | Yes | Yes | 4974 | 156 |
| *63 | 49 | M | White | 12/29/2020 | Yes | Yes | 5876 | 47 |
| 64 | 50 | F | Hispanic or Latino | 11/9/2020 | Yes | Yes | 8088 | 48 |
| GMT | 44 | - | - | - | - | - | 601 | 38 |
| 95%CI | 37-52 | - | - | - | - | - | 405-891 | 29-50 |

* Patients received convalescent plasma treatment.

▿×◇△□○ Patients who gave both 1- and 6-month post-infection sera.

**Supplementary Table 2.** $FFRNT_{50}$ values of 6-month post-infection sera against mNG USA-WA1/2020 and Omicron-spike SARS-CoV-2

| Serum ID | Age | Gender | Race and Ethnicity | Sample collection date yielding positive viral test | Symptomatic | Hospitalized | $FFRNT_{50}$ | |
|---|---|---|---|---|---|---|---|---|
| | | | | | | | USA-WA1/2020 | Omicron-spike |
| ▽ 1 | 17 | F | Hispanic or Latino | 6/22/2020 | Yes | No | 10 | 10 |
| × 2 | 24 | F | Hispanic or Latino | 6/24/2020 | No | No | 10 | 10 |
| ◇ 3 | 24 | F | Hispanic or Latino | 6/24/2020 | Yes | No | 10 | 10 |
| 4 | 70 | M | White | 7/26/2020 | No | No | 10 | 10 |
| 5 | 29 | F | Black or African American | 8/3/2020 | No | No | 18 | 14 |
| 6 | 21 | F | Hispanic or Latino | 6/26/2020 | No | No | 33 | 14 |
| ○*7 | 70 | M | Hispanic or Latino | 12/12/2020 | Yes | Yes | 68 | 21 |
| 8 | 27 | F | Hispanic or Latino | 8/9/2020 | No | No | 69 | 17 |
| 9 | 22 | F | Hispanic or Latino | 10/1/2020 | No | No | 77 | 30 |
| 10 | 61 | F | White | 8/24/2020 | No | No | 82 | 29 |
| 11 | 40 | F | Hispanic or Latino | 7/31/2020 | Yes | No | 85 | 21 |
| 12 | 22 | F | Hispanic or Latino | 7/13/2020 | No | No | 86 | 22 |
| 13 | 50 | F | White | 11/25/2020 | Yes | Yes | 86 | 17 |
| 14 | 26 | F | Hispanic or Latino | 9/10/2020 | No | No | 103 | 22 |
| 15 | 21 | F | Hispanic or Latino | 6/2/2020 | Yes | No | 105 | 10 |
| 16 | 26 | F | Hispanic or Latino | 9/10/2020 | No | No | 106 | 23 |
| 17 | 73 | F | White | 10/14/2020 | Yes | Yes | 125 | 10 |
| 18 | 22 | M | Hispanic or Latino | 9/24/2020 | Yes | Yes | 134 | 27 |
| 19 | 47 | M | Black or African American | 4/23/2020 | No | No | 140 | 14 |
| 20 | 79 | M | White | 5/4/2020 | Yes | Yes | 163 | 44 |
| **21 | 77 | F | Black or African American | 12/7/2020 | Yes | No | 167 | 20 |
| 22 | 57 | M | White | 5/13/2020 | Yes | Yes | 175 | 17 |
| 23 | 23 | F | Hispanic or Latino | 12/25/2020 | Yes | No | 230 | 67 |
| 24 | 40 | F | Hispanic or Latino | 3/16/2020 | Yes | No | 244 | 51 |
| 25 | 22 | F | Hispanic or Latino | 8/11/2020 | Yes | No | 292 | 69 |
| 26 | 54 | M | White | 4/10/2020 | Yes | Yes | 302 | 39 |
| 27 | 64 | M | White | 1/3/2021 | Yes | Yes | 333 | 69 |
| 28 | 39 | F | Black or African American | 7/9/2020 | Yes | No | 337 | 45 |
| 29 | 69 | M | White | 8/14/2020 | Yes | No | 389 | 45 |
| □30 | 49 | M | Black or African American | 1/3/2021 | Yes | Yes | 532 | 55 |
| 31 | 96 | F | White | 12/30/2020 | Yes | Yes | 655 | 86 |
| 32 | 80 | F | Hispanic or Latino | 6/20/2020 | Yes | No | 675 | 85 |

| | | | | | | | | |
|---|---|---|---|---|---|---|---|---|
| 33 | 49 | F | Hispanic or Latino | 3/26/2020 | Yes | No | 719 | 125 |
| 34 | 48 | F | Hispanic or Latino | 6/21/2020 | Yes | Yes | 1059 | 137 |
| ▵ 35 | 66 | M | White | 12/27/2020 | Yes | Yes | 1363 | 162 |
| 36 | 70 | M | Hispanic or Latino | 11/19/2020 | Yes | Yes | 3648 | 554 |
| GMT | 41 | - | - | - | - | - | 142 | 32 |
| 95% CI | 35-49 | - | - | - | - | - | 88-229 | 23-44 |

* Patients received convalescent plasma treatment.

** Patient received therapeutic antibody treatment.

▿ × ◇ ▵ □ ○ Patients who gave both 1- and 6-month post-infection sera.

**Supplementary Table 3.** Primers used for constructing the mNG Omicron-spike SARS-CoV-2.

| Primer Name | Sequence (5'->3') |
|---|---|
| A67V/69-70del-F | GTTACTTGGTTCCATGTTATCTCTGGGACCAATGGTACT |
| A67V/69-70del-R | AGTACCATTGGTCCCAGAGATAACATGGAACCAAGTAAC |
| G142D/143-145del-F | TAATGATCCATTTTTGGACCACAAAAACAACAAAAG |
| G142D/143-145del-R | CTTTTGTTGTTTTTGTGGTCCAAAAATGGATCATTA |
| 211del/L212I/ins214EPE-F | AAGCACACGCCTATTATAGTGCGTGAGCCAGAAGATCTCCCTCAGGGTTTTTCGGC |
| 211del/L212I/ins214EPE-R | GCCGAAAAACCCTGAGGGAGATCTTCTGGCTCACGCACTATAATAGGCGTGTGCTT |
| G339D-F | CTTGTGCCCTTTTGATGAAGTTTTTAACGCC |
| G339D-R | GGCGTTAAAAACTTCATCAAAAGGGCACAAG |
| S371L/S373P/S375F-F | GTCCTATATAATCTCGCACCATTTTTCACTTTTAAGTGTT |
| S371L/S373P/S375F-R | AACACTTAAAAGTGAAAAATGGTGCGAGATTATATAGGAC |
| K417N-F | GGGCAAACTGGAAATATTGCTGATTAT |
| K417N-R | ATAATCAGCAATATTTCCAGTTTGCCC |
| N440K/G446S-F | GAATTCTAACAAGCTTGATTCTAAGGTTAGTGGTAATTAT |
| N440K/G446S-R | ATAATTACCACTAACCTTAGAATCAAGCTTGTTAGAATTC |
| S477N/T478K/ E484A-R | CAATTAAAACCTGCAACACCATTACAAGGTTTGTTACCGGCCTG |
| E484A/Q493R-R | ATATGATCGTAAAGGAAAGTAACAATTAAAACCTGCAAC |
| Q493R/G496S/Q498R/N501Y-F | TTACTTTCCTTTACGATCATATAGTTTCCGACCCACTTATGGTGttgg |
| Q498R/N501Y/Y505H-F | CCGACCCACTTATGGTGTTGGTCACCAACCATACAGAGT |
| T547K-F | CAATGGTTTAAAAGGCACAGGTG |
| T547K-R | CACCTGTGCCTTTTAAACCATTG |
| N679K/P681H-F | CAGACTCAGACTAAGTCTCATCGGCGGGCACGT |
| N679K/P681H-R | ACGTGCCCGCCGATGAGACTTAGTCTGAGTCTG |
| N764K-F | GTACACAATTAAAACGTGCTTTAACTGG |
| N764K-R | CCAGTTAAAGCACGTTTTAATTGTGTAC |
| D796Y-F | CACCACCAATTAAATATTTTGGTGGTTTT |
| D796Y-R | AAAACCACCAAAATATTTAATTGGTGGTG |
| N856K-F | GTGCACAAAAGTTTAAAGGCCTTACTGTTTTGCC |
| N856K-R | GGCAAAACAGTAAGGCCTTTAAACTTTTGTGCAC |
| Q954H-R | GTTTAACAAGCGTGTTTAAAGCTTGTGCATTATGGTTGACC |
| N969K-R | TTTAAAACACTTGAAATTGCACCAAATTTGGAGCTAAGTTGTTTAACAAGCGTGTTT |
| L981F-F | GCAATTTCAAGTGTTTTAAATGATATCTTTTCACGTCTTG |
